# Rare Lymphoma of the Breast: A Case Report

**DOI:** 10.7759/cureus.45059

**Published:** 2023-09-11

**Authors:** Megan Bradley, Katie Kirk, Jayati Mallick, Flavia E Posleman Monetto

**Affiliations:** 1 John Sealy School of Medicine, University of Texas Medical Branch at Galveston, Galveston, USA; 2 Pathology, University of Texas Medical Branch at Galveston, Galveston, USA; 3 Radiology, University of Texas Medical Branch at Galveston, Galveston, USA

**Keywords:** ultrasound, mammogram, radiology, breast cancer, lymphoma of the breast

## Abstract

Lymphoma of the breast is a rare malignancy of the breast lymphoid tissue. It can present as either a primary or a secondary malignancy due to metastasis from a systemic disease. Secondary breast lymphoma (SBL) is one of the most common malignancies to metastasize to the breast. Once present in the breast, these masses are often difficult to distinguish from primary breast carcinoma on both physical examination and diagnostic imaging modalities. Differentiating these tumors is imperative because each has a different management plan. This report presents a rare case of SBL in a 55-year-old Hispanic female and includes a review of its presentation, radiologic imaging findings, and management.

## Introduction

Lymphoma of the breast is a rare tumor, accounting for only 0.4%-0.7% of all breast cancer cases [1]. It can present as either a primary malignancy or a secondary malignancy due to the involvement of the breast from a diffuse disease, with the latter being more common [2]. Secondary breast lymphoma (SBL) is the second most common malignancy to metastasize to the breast, following malignant melanoma [3]. Lesions that metastasize to the breast are often difficult to differentiate from primary breast carcinomas due to their similarities in both physical examination and imaging modalities. As a result, it is imperative to differentiate these tumors by employing a specific diagnostic workup, such as a biopsy, since both malignancies have different treatment plans. Here, we present a rare case of SBL.

## Case presentation

A 55-year-old Hispanic woman presented with a palpable left breast mass localized to the upper outer quadrant in the axillary tail of Spence. Physical exam was remarkable for a yellow discoloration overlying a mobile, palpable two-centimeter mass with no axillary lymphadenopathy. The patient was referred for further radiographic workup including mammography and ultrasound.

**Figure 1:**
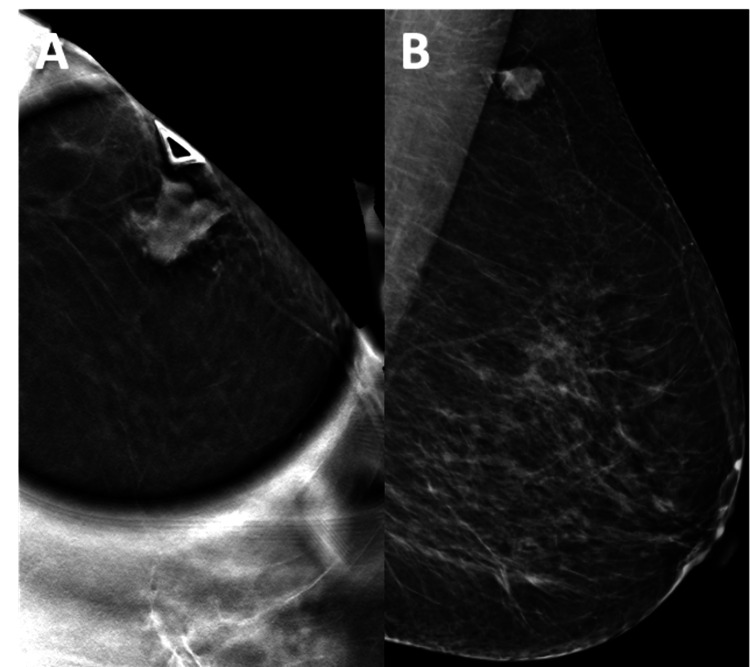
Mammography of the left breast showing a palpable oval mass with indistinct margins in the axillary tail A) 3D craniocaudal (CC) spot compression and B) medial-lateral-oblique view

**Figure 2:**
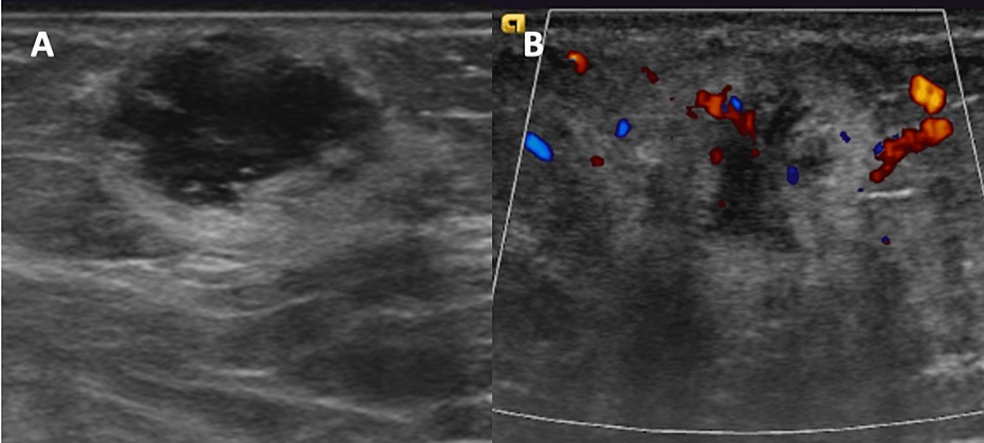
A) Ultrasound of the left breast showing a hypoechoic, irregular mass with irregular margins and B) color Doppler ultrasound showing associated echogenic rim and internal vascularity at the mammographic area of concern

Mammography showed an oval mass with indistinct margins at the one o’clock position, located 13 centimeters from the nipple (Figure 1). The ultrasound demonstrated an oval mass with indistinct margins measuring 16 x 11 x 15 millimeters (Figure 2). No axillary, infraclavicular, supraclavicular, or internal mammary chain lymphadenopathy was noted. The mass was characterized as Breast Imaging Reporting and Data System (BI-RADS) category 4C, and the patient was scheduled for an ultrasound-guided biopsy.

**Figure 3:**
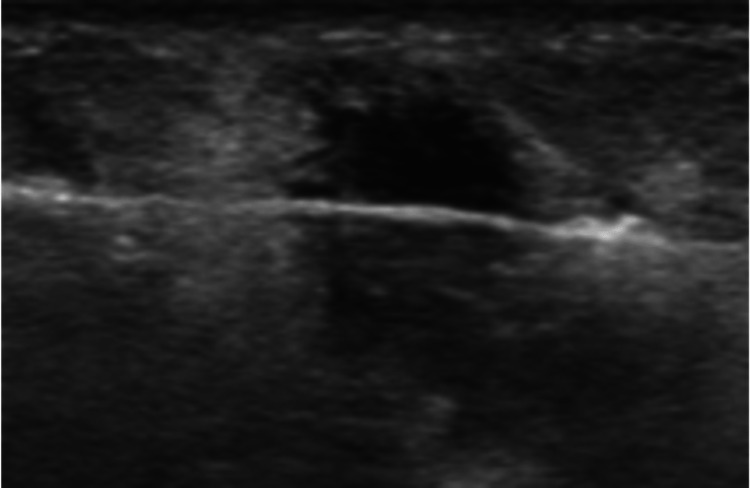
Ultrasound-guided biopsy was performed

**Figure 4:**
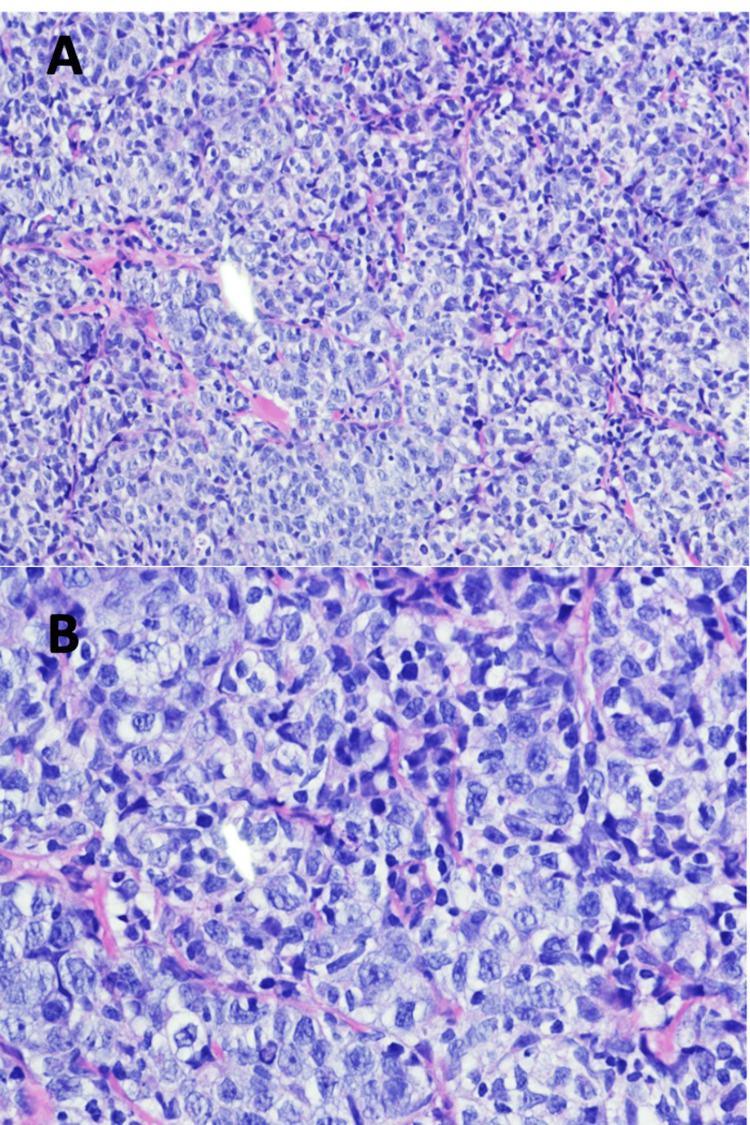
Hematoxylin-eosin (HE stain) stained sections show diffuse proliferation of large, atypical cells with clear cytoplasm, irregular nuclei, vesicular chromatin, and prominent nucleoli A) 200x magnification, B) 400x magnification

**Figure 5:**
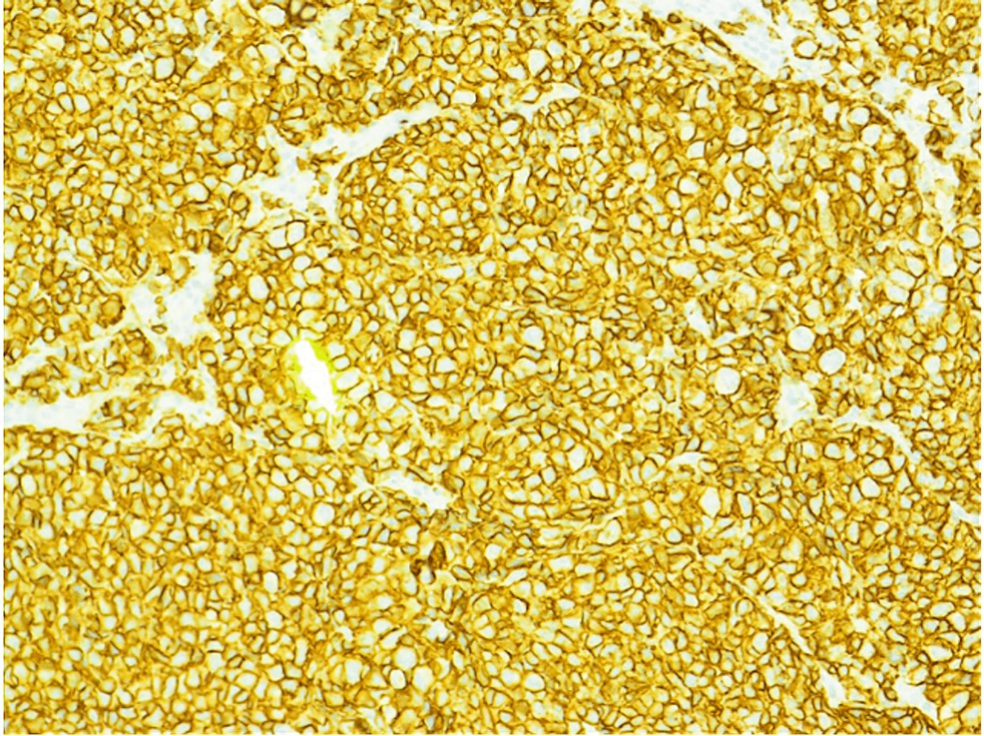
200x magnification showing cells that stain positive for CD-20, a B-cell marker, on immunohistochemistry analysis

**Figure 6:**
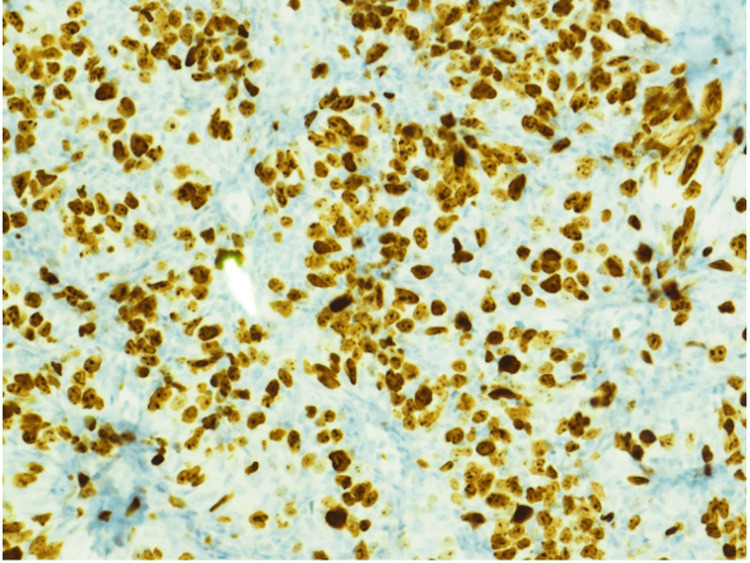
200x magnification of Ki-67 immunohistochemistry analysis showing a proliferative index of 60-70%

Ultrasound-guided biopsy (Figure 3) showed diffuse proliferation of large, atypical cells with clear cytoplasm, irregular nuclei, vesicular chromatin, and prominent nucleoli (Figure 4). Immunohistochemistry analysis demonstrated positive staining for CD-20, a B-cell marker (Figure 5) and Ki-67 staining showed a proliferation index of 60-70% (Figure 6). Further immunohistochemistry analysis showed atypical cells that stained positive for CD-45, BCL-6, and CD-10 but were negative for Her-2-neu, ER, PR, MUM-1, CD-30, ALK, and C-MYC. These findings are consistent with Diffuse Large B-Cell Lymphoma (DLBCL).

**Figure 7:**
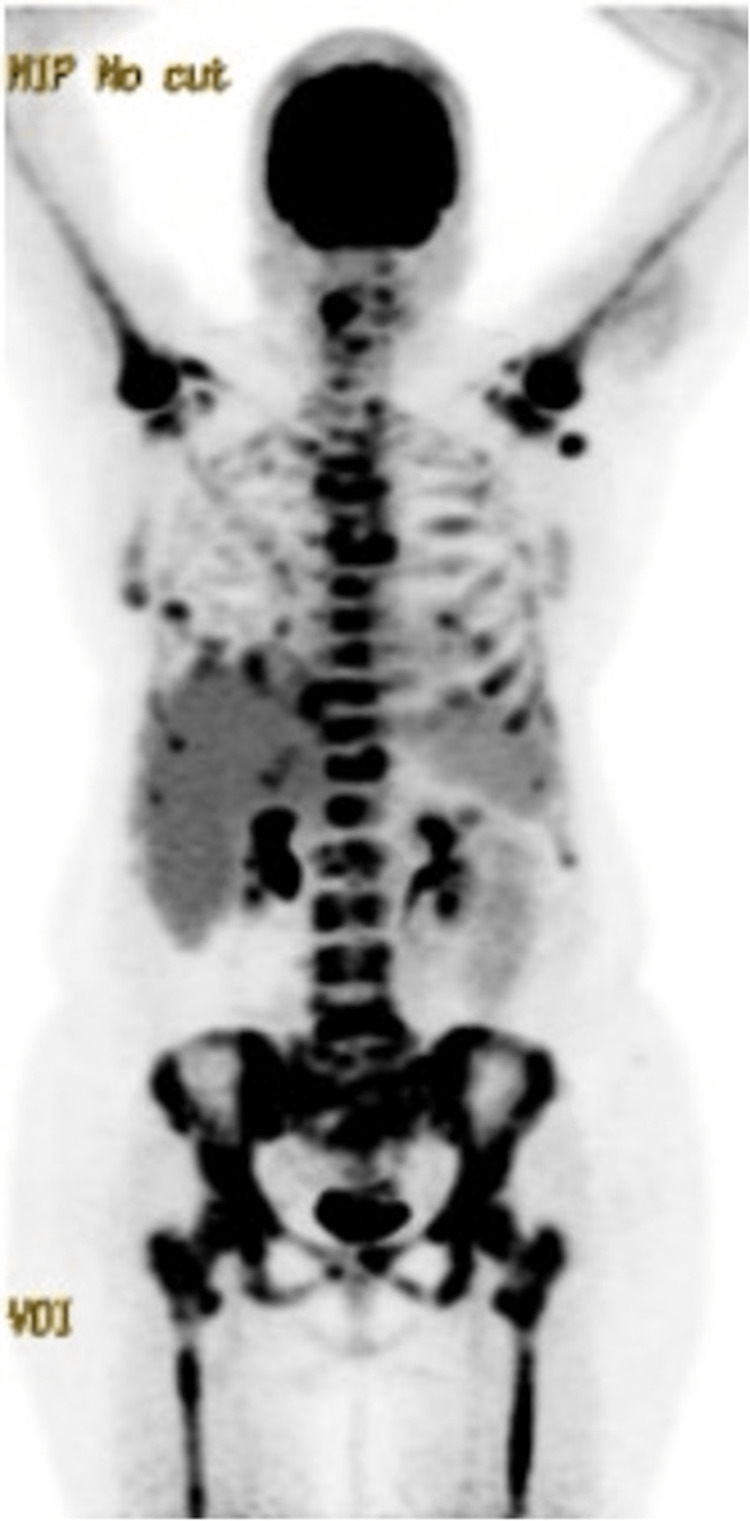
FDG-PET/CT scan showing diffuse osseous involvement of the appendicular and axial skeleton, left axillary lymph node involvement, bilateral hilar lymphadenopathy, bilateral pulmonary spiculated nodules, abdominal lymph nodes (mesenteric, retroperitoneal, and left common iliac chain) involvement, and liver involvement FDG-PET: fluorodeoxyglucose-positron emission tomography

A staging 18F-fluorodeoxyglucose positron emission tomography combined with computed tomography (FDG-PET/CT) (Figure 7) showed diffuse osseous involvement of the appendicular and axial skeleton, a two-centimeter mass of the left breast with ipsilateral axillary lymph node involvement, bilateral pulmonary spiculated nodules with ground-glass opacities, bilateral hilar lymphadenopathy, liver involvement, and abdominal lymph node (mesenteric, retroperitoneal, and left common iliac chain) involvement, consistent with diffuse metastatic disease. The final diagnosis was determined to be stage IV, diffuse large B-cell non-Hodgkin’s lymphoma, and the patient was referred to surgery and oncology.

The patient underwent left heme port placement and completed six cycles of Rituximab, Cyclophosphamide, Hydroxydaunorubicin, Oncovin, and Prednisone (R-CHOP) chemotherapy. Currently, the patient is in remission and continues to have no recurrence, confirmed with a normal mammogram and no suspicious lesions on FDG-PET/CT scan at a two-year follow-up appointment.

## Discussion

Malignant lymphoma of the breast is a rare tumor, accounting for only 0.4%-0.7% of all breast cancer cases and can often mimic the clinical symptoms of breast carcinoma [4]. There are two main subtypes of malignant lymphoma, primary breast lymphoma (PBL) and SBL. PBL occurs when the tumor is localized exclusively to the breast, whereas SBL is diagnosed when other sites of the body have lymphomas, including the breast [5]. This report presented a case of SBL, specifically the DLBCL subtype, which is the most common [6].

The most common symptom of breast lymphoma is a painless, palpable mass located in the outer quadrant [1], similar to breast carcinoma. However, breast carcinoma presents with findings such as peau d’orange and nipple retraction and discharge that are not typically appreciated in breast lymphoma [7]. On mammography, lymphomas often present as an oval or round mass with indistinct margins [8] and are indistinguishable from breast carcinoma through this imaging technique. Since breast lymphomas and breast carcinomas present similarly on physical exams and imaging studies, it is imperative to distinguish these two diagnoses via a more specific modality such as biopsy. Biopsy is considered the gold standard for confirming the proper diagnosis [9] due to its high specificity. Following the biopsy, imaging with fluorodeoxyglucose-positron emission tomography (FDG-PET)/CT should be completed in order to determine staging [10].

Confirming the proper diagnosis is critical because breast lymphoma and breast carcinoma have different treatment plans. For example, breast lymphoma is not usually excised [11], as opposed to breast carcinoma, and the standard treatment is anthracycline-based chemotherapy, with Cyclophosphamide, Hydroxydaunomycin, Oncovin, and Prednisone (CHOP) being the most common [5]. Furthermore, adding Rituximab has been noted to improve survival for these patients [12]. The patient presented in this report received six cycles of R-CHOP chemotherapy and is in continued remission, with no relapse at a two-year follow-up appointment, supporting the efficacy of this treatment plan.

## Conclusions

In conclusion, lymphoma of the breast is a rare case. This diagnosis should be considered in patients who present with suspicious mammogram findings. While mammography provides a sensitive screening method, biopsy must be done because it is more specific for establishing the proper diagnosis. Accurate diagnosis is important since chemotherapy is the treatment of choice for lymphoma of the breast. We hope this case report provides insight into the diagnostic value of both mammography and further workup with biopsy and an FDG-PET/CT scan in order to provide standard-of-care treatment in lymphoma of the breast.
